# The exceptional potential in extending primary care exposure for South African medical interns

**DOI:** 10.4102/safp.v66i1.5911

**Published:** 2024-04-23

**Authors:** Klaus B. von Pressentin, Arun Nair, Shane Murphy, Ramprakash Kaswa, Indiran Govender

**Affiliations:** 1 Division of Family Medicine, Department of Family, Community and Emergency Care, Faculty of Health Sciences, University of Cape Town, Cape Town, South Africa; 2 Department of Family Medicine, University of the Free State, Kimberley, South Africa; 3 Robert Mangaliso Sobukwe Hospital, Northern Cape Department of Health, Kimberly, South Africa; 4 Outpatient Department, Clinical Services, Abbey House Medical Centre, Navan, Ireland; 5 Department of Family Medicine and Rural Health, Walter Sisulu University, Mthatha, South Africa; 6 Mthatha General Hospital, Mthatha, South Africa; 7 Department of Family Medicine and Primary Health Care, School of Medicine, Sefako Makgatho Health Sciences University, Pretoria, South Africa

At the start of 2024, hundreds of medical interns were welcomed nationwide to their family medicine rotation in district healthcare facilities as part of their second internship year. This year marks the fourth year of the extended 6-month internship rotation in family medicine, introduced in January 2021 as part of the revised 2-year internship programme for newly qualified doctors. Interns now spend double the time in district health services compared to their predecessors’ 3-months. This change, considered transformational in training and service delivery, has ensured that smaller district hospitals and primary care facilities can now count interns as part of their teams. The 2-year internship has been in place since 2004, and the 2021 restructuring necessitated that other hospital-based specialities reduce their rotations by 1 month to provide the expansion of the training in family medicine.^1^

Previous research on the value of internship in preparing interns for community service in district hospitals revealed mixed findings, citing supervision issues and dissatisfaction with training facilities’ conditions.^1^ There were also issues of adequate exposure to essential district procedures such as caesarean sections. These findings related mainly to the previous model of the internship programme. However, interns recently captured their reflections on their experience of the revised training programme, citing persisting challenges, including the loss of 1-month specialist learning, unstandardised management of medical emergencies, inadequate orientation and supervision and a need for integrated services.^2^ They called on several stakeholders, including family physicians and intern curators, the Health Professions Council of South Africa (HPCSA) and the Department of Health, to help mitigate these challenges.

These interns’ powerful voices resonate with the findings of a recent exploratory sequential mixed methods study, which zoomed in on the new family medicine rotation for interns in the Western Cape Province.^1^ Interns, managers, supervisors and curators from programmes across central, regional and district hospitals participated in interviews during the study’s first phase. Four major themes emerged, revealing positive experiences and potential benefits such as enhanced care quality, better community service preparation and increased interest in district health services. The subsequent survey phase involving 72 interns and 36 supervisors showcased further positive outcomes such as increased independence and confidence, yet revealed gaps in exposure to community-based services and continuity of care.^3^ While interns and supervisors generally found the rotation appropriate in length, there is a consensus on the need for improvements in exposure, supervision and orientation.

Family medicine departments have a tangible opportunity to use the extended rotation to invest in interns as emerging primary care clinicians who will benefit from leadership skills and, if interested, teaching and research skills. The Academy of Science of South Africa (ASSAf) encourages academic departments to provide additional development opportunities for interns. The 2018 ASSAf consensus report recommended that universities play a more prominent role in internship training by reframing their education and training mandate to include the continuum from the undergraduate years to internship (renamed postgraduate years 1 and 2) and community service (renamed postgraduate year 3).^4^ Furthermore, the HPCSA’s internship guidelines include the need for applied theoretical and academic teaching.^5^ It has specific objectives for public health medicine, such as improving quality of care, critical appraisal of evidence and promoting community health, in the domain of family medicine and primary care.

Realising the exceptional potential of extended primary care exposure for South African medical interns resonates with ethos described in the landmark 1979 paper by Stott and Davis, ‘The exceptional potential in each primary care consultation’. Their framework encourages ‘the primary care physician never to forget the potential of each consultation’ by using a holistic, biopsychosocial approach.^6^ Stott and Davis concluded that

[*T*]he potential of every consultation should be taught to undergraduates and postgraduates, so the next generation of doctors will begin to expect the skills and structures required to carry the special responsibilities of each primary care consultation and then organize their services accordingly.^6^ (p. 205)

The discipline of family medicine and primary care has a profound opportunity to realise the exceptional potential presented by the revised intern programme. An awareness of a person-centred approach to care provision and growth in leadership and clinical governance skills will help interns strengthen and organise health services within their sphere of influence.

The extended family medicine intern rotation can be crucial in better equipping these young professional colleagues for their community service year and potentially a career pathway in primary healthcare.^7^ A study that tracked graduates from one university showed that more than half of their alumni did not go on to specialise, which makes the extended rotation more aligned with most graduates’ career pathways.^8^ However, interns may miss out on this exceptional potential opportunity if used only for menial tasks such as pushing patients to X-rays and putting up drips in under-resourced and poorly managed facilities. District healthcare facilities should provide interns with sufficient supervision and learning opportunities. Family physicians are vital in working with stakeholders to strengthen working conditions and governance in South Africa’s healthcare system to retain these highly skilled professionals post-internship, especially with the national commitment to universal health coverage.^9,10^ This message resonates with the interns’ call for further research and improvement of the internship model to ‘prepare us sufficiently for community health service, strengthen our health system and ultimately achieve health for all those we serve in our communities’.^2^
